# Spinal and Pelvic Alignment of Sitting Posture Associated with Smartphone Use in Adolescents with Low Back Pain

**DOI:** 10.3390/ijerph18168369

**Published:** 2021-08-07

**Authors:** Tae-sung In, Jin-hwa Jung, Kyoung-sim Jung, Hwi-young Cho

**Affiliations:** 1 Department of Physical Therapy, Gimcheon University, Gimcheon 39528, Korea; 20160072@gimcheon.ac.kr; 2 Department of Occupational Therapy, Semyung University, Jecheon 27136, Korea; otsalt@semyung.ac.kr; 3 Department of Physical Therapy, College of Health Science, Gachon University, Seongnam 13120, Korea

**Keywords:** low back pain, sitting, kyphosis, lordosis

## Abstract

This study aimed to assess the association between smartphone use in the sitting posture and changes in thoracolumbar kyphosis, lumbar lordosis, and pelvic asymmetry in adolescents with low back pain (LBP). Twenty-five adolescents with LBP and 25 healthy adolescents participated in this study. They were instructed to sit on a height-adjustable chair with their hips and knees bent at 90° for 30 min in their usual sitting postures. Thoracolumbar kyphosis, lumbar lordosis, and pelvic asymmetry were measured using a three-dimensional motion capture system. Thoracolumbar kyphosis and lumbar lordosis increased after 30 min of sitting compared to the baseline. In both groups, thoracic kyphosis and lumbar lordosis angle increased with increasing sitting time. Compared to healthy adolescents, adolescents with LBP presented greater thoracolumbar kyphosis and lumbar lordosis after prolonged sitting. Pelvic asymmetry showed no significant difference between the sitting time and groups. Using a smartphone during prolonged sitting may lead to a slumped posture; these associations were more pronounced in adolescents with LBP.

## 1. Introduction

Since 2007, smartphones have rapidly penetrated the daily lives of adolescents and have become the most basic item for performing daily activities such as listening to music, browsing the Internet, watching videos, and taking pictures [1]. Over the past 10 years, both the time and frequency of smartphone use have increased rapidly [2]. In 2018, 95% of youngsters in the United States reported owning or accessing a smartphone, and 45% of teenagers were almost constantly online [3]. All parts of the body, including the head, neck, shoulders, wrists, hands, and trunk, are used while using a smartphone. It has been reported that muscle activity and muscle fatigue of the neck extensors increase as the flexion angle of the neck increases during smartphone use [4,5]. A study on the spinal posture during smartphone use in a standing posture showed that thoracic kyphosis and trunk inclination increased during all tasks [6]. In a review study on smartphone use, it was found that neck flexion angle increased more in the sitting posture than in the standing posture, and the use of a smartphone could cause musculoskeletal pain [7].

People spend approximately 6 h a day on average in a sitting posture, and 37% of them sit for >8 h [8]. In addition, adolescents spend much of their day in a sitting posture because of their schoolwork [9]. Lumbar lordosis is decreased more in the sitting position than in the standing position [10,11]. This change in posture increases the weight and stress on the lumbar spine and surrounding structures, and it increases the risk of low back pain (LBP) [12]. The various muscles of the trunk, commonly known as core muscles, are very important musculature for performing various movements or maintaining postures in daily life, especially for tasks performed while sitting on a chair, maintaining endurance, and providing stability to the trunk muscles [13]. Previous studies have reported that various musculoskeletal disorders are associated with maintaining an uncomfortable or inappropriate sitting posture for a long time [14,15,16]. The age at which such LBP occurs is gradually decreasing. In the 2000s, it was reported that LBP appeared in the early teens, with one-third of 14-year-olds complaining of back pain [17,18].

Although proper upright sitting posture involves anterior pelvic tilt, lumbar lordosis, and relaxation of the thorax [13,19], which reduces the pressure on the intervertebral discs [20], most people tend to sit in a slouched posture for prolonged durations, which is often accompanied by a deformed posture in the spine and pelvis [21]. When the posture is maintained with the neck or trunk bent forward, the back extensors are over-activated, and the constant load on these muscles may increase the risk of neck and back pain [22,23]. Hey et al. [21] analyzed the natural sitting postures of patients diagnosed with LBP and reported increased cervical lordosis and thoracic kyphosis as well as decreased lumbar lordosis, compared to the upright sitting posture. Furthermore, in previous studies comparing the sitting postures of LBP patients and healthy individuals, the former showed an increase in the lumbar flexion angle [24] and a decrease in hip flexion and trunk muscle endurance [25] compared with the latter.

Each vertebra of the spine is a separate structure but is connected by a kinematic chain from the neck to the sacrum; thus, the use of smartphones in various postures either increases head flexion or changes the alignment of the thoracic and lumbar spine [6]. However, most studies examining the effects of smartphone use on the human body have focused on the neck, and there are still few studies that have investigated the effects of smartphone use on the changes in other vertebrae. Adolescence is a period of significant physical, emotional, cognitive, and behavioral developmental changes [26]. To our knowledge, there are no studies that have investigated the association between smartphone use in a sitting position and changes in the alignment of the spine and pelvis, and it is necessary to determine whether these changes differ depending on the presence or absence of LBP.

Jia and Nussbaum’s study [27] showed that in healthy adults, maintaining a sitting position for at least 30 min could induce muscle fatigue. Therefore, the present study aimed to assess the association between smartphone use during prolonged sitting and changes in thoracolumbar kyphosis, lumbar lordosis, and pelvic asymmetry in adolescents with and without chronic LBP.

## 2. Subjects and Methods

### 2.1. Participants

This study included 25 adolescents (16 males and 9 females) with non-specific LBP and 25 healthy adolescents (15 males and 10 females, mean age: 17.98 ± 0.68) residing in G city of the Republic of Korea. The inclusion criteria were (1) non-specific LBP for ≥3 months without specific pathologies such as infection, tumor, osteoporosis, fracture, structural deformity, an inflammatory disorder, radicular syndrome, or cauda equina syndrome [28]; (2) age 10–19 years; (3) pain intensity ≥ 3 on the visual analogue scale; and (4) 1 or more hours of smartphone use every day for at least 1 year [6]. The exclusion criteria were as follows: (1) referred pain down to the leg; (2) use of analgesics before the baseline test; (3) fracture or surgery in the spine within the past 2 years; and (4) musculoskeletal pain in other areas such as the shoulder and neck [29]. The control group participants had no previous episodes of LBP in the last 6 months and showed no abnormal findings on radiological or physical examinations, including testing of reflexes and superficial sensation, a motor and sensory test, and a straight leg raising test.

The study was approved by the Institutional Review Board of Gachon University (1044396-202004-HR-078-01) and was performed according to the specified protocol. All individuals voluntarily participated in the study. The study was explained to them by the researchers, and signed informed consent was obtained before participation.

G*power 3.1.9.4 software (Heinrich-Heine-University Düsseldorf, version 3.1.9.4, Düsseldorf, Germany) was used to calculate the sample size. In the present study, the mean power and the alpha error were set at 0.8 and 0.05, respectively. In addition, the effect size was set to 0.7669650 based on a pilot study (10 participants). The analysis of the G*power software showed that at least 22 participants were recruited to make an acceptable group sample size for each group; thus, 50 participants were recruited after considering drop-out.

To recruit participants, we displayed a poster describing the purpose of the study (influence of smartphone use on changes in the spinal and pelvic alignment in adolescents with and without LBP) and the inclusion and exclusion criteria on the bulletin boards and websites of local high schools and private institutes in G city. One researcher received the individual applications of potential subjects and checked whether they met the inclusion and exclusion criteria. The researcher notified the applicants the next day regarding whether they could participate in the study, and then they introduced the experiment schedule and factors to be limited (e.g., food intake and toilet use) during the research process.

### 2.2. Protocol

Participants were seated on a chair without desks or backrests to perform a 30 min mobile device usage task. The height of the chair was adjusted such that each participant’s knee was flexed at 90° before the measurements were performed. The researchers instructed the participants to place their arms next to their trunks while using a smartphone without mechanical assistance, such as a brace or weight support device, and requested the subjects to type in messengers and social networking services using both hands in the usual way. Since this study aimed to investigate the association between smartphone use and the subject’s postural alignment, all types of smartphone use such as sending a text message, listening to music, searching the Internet, and watching videos were allowed. During the smartphone task, the smartphone was allowed to be used at the participant’s preferred position without any restrictions [6]. At the start of the 30 min sitting period, the participants were instructed to maintain a comfortable but upright sitting posture. One minute after the initiation of the measurement, participants were instructed to perform the task in any comfortable position, except for cross-legged sitting. The participants were not aware of the exact measurement time and were asked to perform the task until a beep sound was heard. During the 32 min task time, kinematic data were recorded. To compare postural changes, data related to the first and last minute were analyzed.

**Figure 1:**
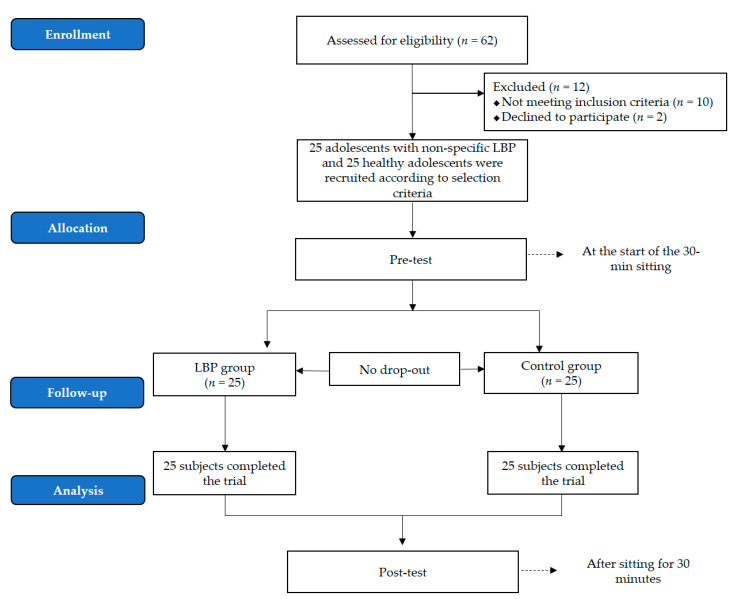
Flow diagram.

Before the actual measurement, all subjects practiced to become familiar with the task. Of the 62 recruited participants, 12 did not meet the inclusion criteria or refused to participate; therefore, 25 people for each group finally participated in the experiment, and there were no drop-outs (Figure 1).

### 2.3. Outcome Measurements

We assessed thoracolumbar kyphosis, lumbar lordosis, and pelvic obliquity using a motion capture system with 10 infrared cameras (Raptor-E, Motion Analysis Inc., Rohnert Park, CA, USA). Kinematic data were obtained and analyzed using video motion analysis software (ORTHOTRAK6.2.4, Motion Analysis Inc., Rohnert Park, CA, USA). The sampling frequency was set to 100 Hz.

To measure spinal curvatures, markers were attached to the spinous processes (SPs) of the T1, T5, T10, L3, and S2 vertebrae.

The thoracolumbar kyphosis angle was measured based on the angle between the line connecting the SPs of T5 and T10 vertebrae and that connecting the SPs of T10 and L3 vertebrae. Lumbar lordosis was measured by the angle between the line connecting the SPs of T10 and L3 and that connecting the SPs of L3 and S2 [30].

To obtain pelvic asymmetry, markers were attached to the bilateral anterior superior iliac spines (ASISs). Pelvic asymmetry in the frontal plane, commonly known as pelvic obliquity, presents as one innominate bone higher or lower than the other [31]. Pelvic obliquity was measured according to the angle formed by the horizontal line and the line connecting both ASISs [32].

### 2.4. Data Analysis

SPSS 21.0 (IBM, Armonk, NY, USA) was used for the statistical analysis. The normality of the variables was assessed using the Shapiro–Wilk test. The independent *t*-test for continuous variables and the chi-square test for categorical variables were used to compare the subjects’ general characteristics between the LBP and control groups. The effects of time and group and their interaction on thoracolumbar kyphosis, lumbar lordosis, and pelvic obliquity were examined using two-way repeated-measures ANOVA. When a significant interaction between independent variables was detected, a paired *t*-test was used to investigate the effect of time in each group. The level of statistical significance was set at *p* < 0.05.

## 3. Results

### 3.1. General Characteristics of the Subjects

Thirty-five adolescents with LBP and 27 healthy adolescents without LBP voluntarily applied for the study. Twelve subjects were excluded for the following reasons: 10 LBP adolescents did not meet the selection criteria (two were taking pain medication, five were diagnosed with scoliosis, and three did not have enough time to use a smartphone), and two healthy adolescents did not follow the study protocol for personal reasons. Table 1 shows the general characteristics of the participants in the two groups. No significant difference in any variable was noted between the two groups. The average duration of pain in the LBP group was 8.56 ± 1.96 months; 88% of patients (22 patients) with LBP reported that the pain worsened in the lumbar flexion posture, and the remaining three patients reported that the pain worsened during lumbar extension. There was no difference between the groups in thoracolumbar kyphosis, lumbar lordosis, and pelvic asymmetry in an upright sitting posture before they performed the task for 30 min (*p* < 0.05).

### 3.2. Comparison of Thoracolumbar Kyphosis

**Figure 2:**
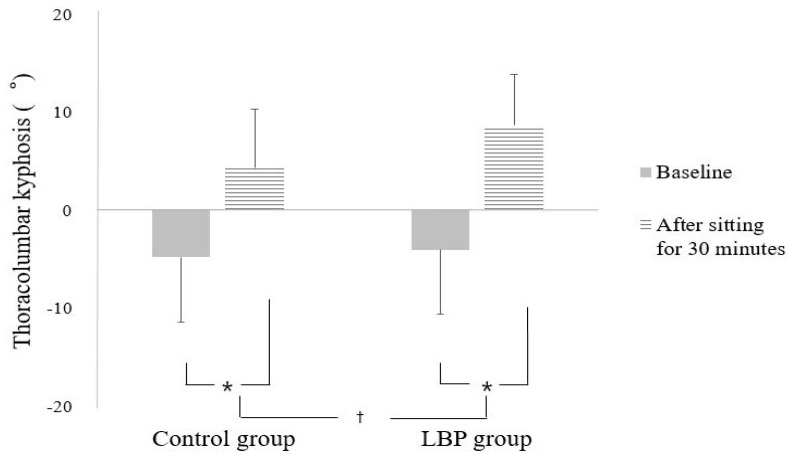
Mean of thoracolumbar kyphosis before and after sitting. * Significant differences between pre and posttest (*p* < 0.05). † Significant differences between groups (*p* < 0.05).

There were significant differences between the two groups in the change in thoracolumbar kyphosis angle according to sitting time (interaction effect between group and time: F = 5.871, *p* = 0.019) (Figure 2).

Thus, follow-up analyses were performed using *t*-tests to investigate the effects of sitting time within each group.

Simple main effect analyses of the thoracolumbar kyphosis angle revealed that the thoracolumbar kyphosis angle after sitting for 30 min was significantly increased in both groups compared to the initial value (t = −12.375, *p* < 0.001, effect size: 2.11, for LBP group; t = −8.833, *p* < 0.001, effect size: 1.25, for control group) (Figure 2).

### 3.3. Comparison of Lumbar Lordosis

**Figure 3:**
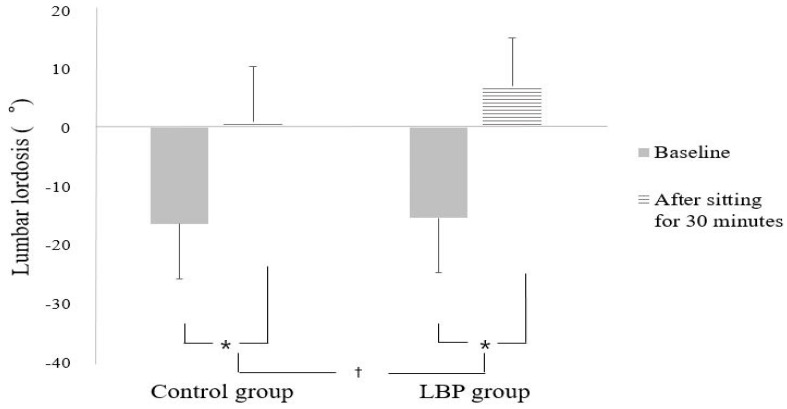
Mean of lumbar lordosis before and after sitting. * Significant differences between pre and posttest (*p* < 0.05). † Significant differences between groups (*p* < 0.05).

There were significant differences between groups in the change in lumbar lordosis angle according to sitting time (interaction effect between group and sitting time: F = 6.531, *p* = 0.014) (Figure 3).

Thus, follow-up analyses were performed using *t*-tests to investigate the effects of sitting time within each group.

The simple main effect analyses of the lumbar lordosis angle revealed that the lumbar lordosis angle after sitting for 30 min was significantly increased in both groups compared to the initial value (t = −15.982, *p* < 0.001, effect size: 2.54, for LBP group; t = −12.806, *p* < 0.001, effect size: 1.61, for control group) (Figure 3).

### 3.4. Comparison of Pelvic Asymmetry

**Figure 4:**
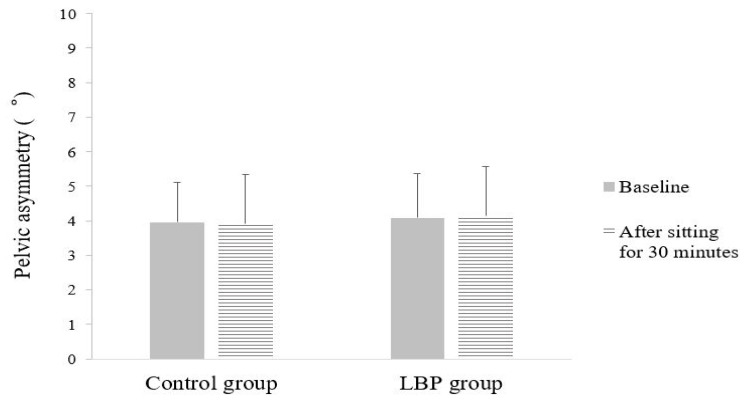
Mean of pelvic asymmetry.

There were no significant differences between groups in the change in pelvic asymmetry according to sitting time (interaction effect between the group and sitting time: F = 0.488, *p* = 0.486) (Figure 4).

## 4. Discussion

In this study, we investigated the association between extended smartphone use in a sitting position and the change in thoracolumbar kyphosis angle in adolescents with and without LBP. The movement of the thoracic spine is limited by the thoracic structure, and it shows a smaller angle change compared to the lumbar spine with various movements and postures [21]. Nevertheless, according to our results, the thoracolumbar kyphosis angle significantly increased after smartphone use in a sitting position for 30 min in both groups, and the LBP group showed a greater change in this trend than the control group. The smartphone addiction rate among adolescents and adults was reported to be 1.0–9.3% [33], and many studies have been conducted on the negative effects of smartphone use on the neck and spine [4,5,34]. Betsch et al. reported that using a smartphone while standing or walking increases thoracic kyphosis and trunk inclination, and this tendency increases with active tasks such as sending text messages, rather than with passive tasks such as simply talking on the phone [6]. In addition, studies on the influence of neck flexion angle on smartphone use have shown that the activation and fatigue of the neck muscle increases as the neck flexion angle increases [4,5]. According to a review, smartphone use intensified slumped posture in a sitting position, rather than a standing position, and caused the spine and shoulders to bend forward, suggesting that excessive smartphone use could cause musculoskeletal disorders [7]. In this study, we compared the effect of smartphone use on posture in the sitting position according to the presence or absence of LBP. A significant change was found in both groups, but the posture change was greater in adolescents with LBP. This result could be due to decreased muscle endurance of the trunk associated with LBP. A study comparing muscle endurance of trunk muscles according to back pain found that adolescents with LBP had significantly decreased trunk muscle endurance compared to healthy adolescents [35]. One study that analyzed the sitting posture of patients with LBP found that the posture of these patients was more slumped than that of healthy people [21]. Waongenngarm et al. reported that sitting in a slumped position for a long time induces trunk muscle fatigue [36]. Furthermore, increased inclination of the head and trunk increases the load on the cervical or lumbosacral joint, leading to neck pain or LBP [37].

Moreover, this study showed a significant difference in lumbar lordosis between the two groups during smartphone use for 30 min in the sitting posture. In general, lumbar lordosis while sitting in an erect posture is reduced by approximately 50% compared to the standing posture. In addition, the subjects had an approximately 80% reduction in lumbar lordosis in the natural sitting position compared to sitting in an erect posture [8,38]. In a study that analyzed the effect of LBP on the sitting posture, patients complaining of pain during trunk flexion in the LBP group showed a decreased tendency of lumbar lordosis than healthy individuals [39]. Likewise, in this study, >88% of the participants in the LBP group had worsening pain during trunk flexion. Hence, it is thought that lumbar lordosis in this group decreased compared to that in the control group during prolonged sitting. Dankaerts et al. argued that this postural change occurs not because of a reflexive response to pain but because of a decrease in postural control ability [39]. Several studies have reported that the proprioception of LBP patients decreases [40]; in patients whose pain worsens during trunk flexion, the activity of the local stabilizing muscle of the trunk was reduced [41]. Therefore, it is assumed that patients with LBP have increased tissue deformation and damage due to a decrease in proprioceptive sense and trunk control ability [42].

In certain cases, postural and structural asymmetry might contribute to LBP; in particular, the association between pelvic asymmetry and LBP is high [43,44]. In a study that measured trunk motion asymmetry in a sitting posture in patients with LBP and healthy individuals, there was a significant difference between the groups. They argued that this difference occurred because the alignment of the spine is altered to compensate for the asymmetry of the pelvis in patients with LBP [45]. However, unlike these studies, our results showed no significant change in pelvic asymmetry in the LBP group during the task in the sitting position for an extended period. In a study comparing pelvic obliquity in standing posture in LBP patients and healthy adults, no significant difference was found between the groups, and various factors other than pelvic asymmetry influenced LBP [29]. Furthermore, Snijders et al. reported that many people adopt a cross-legged posture when sitting for long periods at work and in multiple settings [46]. A cross-legged sitting posture has been reported to increase pelvic obliquity compared to an upright sitting posture [47]. However, in this study, since there was a concern that the variation among the participants would become too large, subjects were instructed to take any posture other than the cross-legged sitting position during measurement. In this study, the lack of a significant difference in pelvic asymmetry between the groups could be due to this limitation of the patients’ sitting postures. Additionally, pelvic asymmetry indicates that the alignment in the frontal or sagittal plane is asymmetric with respect to the vertical axis [48]. However, in this study, only the pelvic asymmetry in the frontal plane was measured, and the change in the pelvic asymmetry in the sagittal plane was not confirmed; therefore, this should be investigated in future studies.

This study compared postural changes, such as alignment of the spine and pelvis, with smartphone use during prolonged sitting in adolescents with or without back pain. The results were similar to those of previous studies, and it was found that smartphone use in a sitting position induced a tendency to increase thoracic kyphosis and lumbar lordosis in the subjects [6,21]. However, this study has several limitations. First, the subjects of this study were mostly in their late teens; therefore, the representativeness of the results is limited to adolescents. Second, it is difficult to generalize the results due to the small sample size and confounding factors that may affect LBP, such as exercise and sedentary behaviors, other than smartphone use. Third, as the neck angle and shoulder asymmetry were not measured, the overall effect of smartphone use on sitting posture was not confirmed. Finally, the case–control design of this study allows only to assess associations, and it prevents any conclusion on causality or on the effect of smartphone use on LBP. Further studies with the appropriate design are needed to assess the effect of long-term smartphone use on back pain and its clinical relevance.

## 5. Conclusions

This study found that thoracolumbar kyphosis increases during smartphone use, and this increase may be larger in adolescents with LBP.

## Figures and Tables

**Table 1:** General characteristics of the subjects.

Variables	LBP Group (*n* = 25)	Control Group (*n* = 25)	*p*
Sex (male/female)	15/10	17/8	0.771 ^b^
Age (years) ^a^	17.96 ± 0.73	18.00 ± 0.65	0.839 ^c^
Height (cm) ^a^	171.68 ± 5.93	172.52 ± 8.43	0.685 ^c^
Weight (kg) ^a^	67.36 ± 14.12	69.04 ± 11.02	0.641 ^c^
VAS (score) ^a^^,d^	4.12 ± 0.83	-	
Postures that make symptoms worse (lumbar flexion/extension)	22/3	N/A	

^a^ Mean ± standard deviation, ^b^ Chi-square test, ^c^ Independent *t*-test, ^d^ Score range: 0–10. LBP, low back pain; VAS, visual analogue scale.

## Data Availability

The data presented in this study are available on request from the corresponding author.
